# Evaluation of antenatal simulation-based learning on satisfaction and self-confidence levels among Thai undergraduate nursing students during the COVID-19 pandemic: a mixed-method study

**DOI:** 10.1186/s12912-024-01824-0

**Published:** 2024-03-06

**Authors:** Kornkanok Kuesakul, Sasitara Nuampa, Rudee Pungbangkadee, Lucie Ramjan, Ameporn Ratinthorn

**Affiliations:** 1 Department of Obstetrics and Gynecological Nursing, Faculty of Nursing, Mahidol University, Bangkok, 10700 Thailand; 2 School of Nursing and Midwifery, Western Sydney University, Locked Bag 1797, Penrith, NSW 2751 Australia

**Keywords:** Simulation, Antenatal care, Satisfaction, Self-confidence, Nursing student

## Abstract

**Background:**

During the COVID-19 pandemic, simulation-based learning (SBL) serves as an alternative teaching strategy for nursing students facing restricted access to antenatal clinical practicum. However, the factors predicting nursing students’ satisfaction, self-confidence, and their learning experiences remain unclear.

**Objective:**

To identify factors predict satisfaction and self-confidence and explore the learning experiences of antenatal SBL.

**Methods:**

A Mixed methods research of the cross-sectional study design and descriptive qualitative research was conducted. A total of 100 third year nursing students who finished the Maternity-Newborn Nursing and Midwifery Practice course using antenatal simulation-based learning were invited to complete the online questionnaires. A total of seven questionnaires were administered, including a demographic questionnaire, the Attitude Scale toward Simulation-Based Education (SBE), the Professional Identity Scale for Nursing Students, the Perceived Stress Scale, the Evaluation of Teaching Competencies Scale, the Simulation Design Scale: Student Version, and the Student Satisfaction and Self-Confidence in Learning. The 20 nursing students who completed survey were asked to participate a qualitative focus group discussion. Multiple regression analysis was performed to investigate predictors, while qualitative data were analyzed using content analysis.

**Results:**

The quantitative results showed high levels of satisfaction (mean = 20.55, SD = 3.17) and self-confidence (mean = 32.44, SD = 4.76) after completing the antenatal SBL. In regression analysis, attitude toward SBE (Beta = 0.473, *t* = 5.376, *p* < 0.001) and attitude toward antenatal care simulation design (Beta = 0.338, *t* = 2.611, *p* < 0.011) were significantly associated with a high level of satisfaction with antenatal SBL, which accounted for 44.0% of the variance explained in satisfaction. Only attitude toward SBE was significantly associated with a high level of self-confidence in antenatal SBL (Beta = 0.331, t = 3.773, *p* < 0.001), which accounted for 45.0% of the variance explained in self-confidence. The qualitative results generated four themes: (1) positive attitude toward antenatal simulation; (2) turning reassurance into confidence; (3) I am really happy to learn; and (4) being a good nurse motivates and stresses me.

**Conclusions:**

Antenatal SBL is an effective teaching strategy that can support nursing students to build clinical confidence. Creating a positive learning environment allows students to have a positive attitude and experience with simulations.

## Introduction

The COVID-19 pandemic forced many nursing schools to become virtual. Clinical placement experiences transitioned to virtual simulation-based learning (SBL) experiences or reduced hours in the clinic. Simulation-based and virtual education experiences allowed students to complete their education and meet regulatory requirements and supported the future of the nursing workforce [1]. Undergraduate nursing programs very quickly transitioned from in-person, face-to-face, and clinical learning to remote and virtual simulation learning during the pandemic [2, 3].

Simulation helped students enrolled in undergraduate healthcare education understand the theoretical principles and practice key skills in a controlled environment [4]. The integration of simulation with clinical placements helped students practice clinical behaviors and skills in a safe environment. This enhanced their confidence and encouraged them to transfer their learning to actual care situations in clinical practice [4, 5]. SBL had benefits and was used as a substitute for nursing clinical practice [6] and pre-clinical simulation-based training among undergraduate nursing students [7]. The pre-clinical simulation could also increase students’ knowledge, skill competencies, confidence, and satisfaction [7].

Learning through simulation is advantageous for several reasons. It allows for repeated experiences, practice, and individual learning, as well as providing immediate feedback [8]. SBL has been shown to consistently increase satisfaction with learning among nursing students [9–11] and promote self-confidence and clinical competence [11, 12]. A previous study showed that students who practiced in simulation workshops perceived confidence in performing health teaching and were successful in clinical practice [13].

Recent studies have explored essential factors related to student confidence and satisfaction according to the National League for Nursing/Laerdal Jeffries Simulation Theory which composed of educational practices, facilitators, participants, simulation design characteristics, and expected outcomes [14]. The framework is useful for developing, implementing, and evaluating simulation-based activities in nursing education. Factors that affect learning satisfaction and self-confidence in SBL included personal factors such as previous learning outcomes [15], attitude toward SBL [16, 17], professional identity [18, 19], perceived stress [20, 21], and facilitator factors; teaching competencies [11, 22]; and simulation design factors [23].

Care management during pregnancy is essential for ensuring the quality of care [24]. With the increase in the constraints of real-world situations, antenatal SBL may be an effective approach to achieving learning outcomes among students. Therefore, effective training with simulation is needed for nursing students to develop the competency to care for pregnant women and achieve learning outcomes. However, few studies have explored the factors associated with nursing students’ confidence and satisfaction related to simulation-based learning in antenatal nursing care. Moreover, nursing students’ experiences and perceptions of the transition from clinic to virtual antenatal simulation training during the COVID-19 pandemic are underexplored.

In contrast to previous studies, our research introduces a novel perspective on nursing practicum training by emphasizing the significance of antenatal SBL and optimizing student learning outcomes amid restricted resources during COVID-19 through a comprehensive understanding.

To assess the usefulness of antenatal simulation in preparing future nurse professionals, we aimed to examine the factors influencing nursing students’ satisfaction and self-confidence levels following antenatal SBL. Additionally, this study aimed to explore nursing students’ learning experiences to better understand their perspectives after completing the antenatal SBL.

## Materials and methods

### Design

This study was part of a larger study, “The Study of Practicing Learning Outcomes from Clinical Simulation in Maternity-Newborn Nursing and Midwifery Practicum among Nursing Students.” The mixed-methods design followed an explanatory sequential approach [25]. First, a cross-sectional survey was administered to evaluate the factors influencing nursing students’ satisfaction and self-confidence levels in antenatal SBL during the COVID-19 pandemic. This was followed by a qualitative study in which focus group interviews were conducted to explore the experiences and perspectives of students who completed antenatal SBL.

### Participants and setting

The sample size was calculated using the G-power 3.1.9.4 software with the following command for linear multiple regression, effect size f^2^ 0.15 (medium size) [26], an alpha level of 0.05, a power of 0.80, and six predictors. Consequently, the calculated sample size is 100 cases. A total of 100 nursing undergraduates from the third year of nursing students who completed the Maternity-Newborn Nursing and Midwifery Practice course and 3 credit hours of clinical work. Inclusion criteria included (1) full-time Thai national students, (2) not previously enrolled in the Maternity-Newborn Nursing and Midwifery Practice course, and (3) having a smartphone or other electronic device to complete the online questionnaire.

This study was conducted at an urban state university in Bangkok involving students enrolled in a baccalaureate nursing program. The antenatal simulation is the clinical part of the Maternity-Newborn Nursing and Midwifery Practice course. In this course, antenatal practical skills for nursing students consisted of (1) an antenatal physical and mental assessment, (2) an abdominal examination for pregnant women over 28 weeks of gestation, and (3) counseling for promoting healthy pregnancy and managing common discomforts. All simulation scenarios were designed and validated by the instructor team according to the course learning outcomes. The onsite simulation was held at the Learning Resource Center. Before starting the scenario, a pre-briefing was conducted by the facilitator to inform the participants of the objectives and rules for simulation learning. During the simulated scenario, other students and the facilitator acted as observers. At the end of the scenario, the facilitator debriefed the students to provide suggestions for improvement and allow them to reflect on their experiences and feelings after completing the scenario.

### Data collection

Data were collected between May and December 2022. After finished the Maternity-Newborn Nursing and Midwifery Practice course, nursing students who were recruited into this study and signed consent forms (*n* = 100) were sent the links to an online cross-sectional survey. They were asked to complete them within 48 h. Next, nursing students who completed online survey were asked to participate in a 45–60-minute qualitative focus group discussions (FGDs). Twenty students were separated into four FGDs (five student per group) depended on the satisfaction levels. There were two groups of low-high level of satisfaction scores.

Semi-structured FGDs were conducted by a video call to explore perceptions and experiences related to antenatal SBL during COVID-19. Field notes and a codebook were used for the analysis.

### Ethical approval

The study was conducted in accordance with the Declaration of Helsinki and approved by the Institutional Review Boards (or Ethics Committees) of the Faculty of Nursing, Mahidol University (COA No. IRB-NS2022/666.1702). The online consent forms were administrative prior data collection.

### Measures

A self-administered online survey consisting of three sections was sent to the students via three different links. A total of seven questionnaires were administered as shown in Table 1. Moreover, a 15-question semi-structured focus group guide was used to collect students’ experiences of the antenatal simulations, which included the following questions: How did you feel after the practice simulation in antenatal care? What were your impression/thoughts about the simulation? What would you change? What were your expectations/goals? How did you achieve them?

**Table 1:** List of self-administered online questionnaires

Measurement	Specific measure	Cronbach’s alpha, this study
Section 1		
Demographic questionnaire	Age, Gender, Cumulative GPA, Maternity-Newborn Nursing and Midwifery lecture grade, SBL experience	N/A
Attitude scale toward simulation-based education (SBE)	18 items, 5-point Likert scales ranging from 1 (strongly disagree) to 5 (strongly agree), range 18–90, a higher score indicating a high level of attitude toward SBE. Demonstrated reliability and validity [17].	0.708
Professional identity scale for nursing students	17 items, 5-point Likert scales ranging from 1 (strongly disagree) to 5 (strongly agree), range 17–85, a higher score indicating a high level of positive perception of professional identity. Demonstrated reliability and validity [27].	0.876
Section 2		
Perceived stress scale	10 items, 5-point Likert scales ranging from 0 (almost never) to 5 (almost always), range 0–50. 1–13 scores indicate a low level of stress, 14–26 indicate a mild level of stress, and 27–40 scores indicate a high level of stress. Demonstrated reliability and validity [28, 29].	0.853
Evaluation of teaching competencies scale	9 items, 3-point Likert scales ranging from 1 (disagree) to 3 (agree), range 9–27, a higher score indicating a high level of positive perception of teaching competencies. Demonstrated reliability and validity [30].	0.882
Simulation Design Scale: student version	Two parts of the questionnaires were attitude toward simulation design (19 items) and perceived important design (19 items), 5-point Likert scales ranging from 1 (strongly disagree) to 5 (strongly agree), range of 19–95 for each part, a higher score indicating participants perceived the design of ANC simulation with a high degree of suitability and importance. Demonstrated reliability and validity [31].	0.969, 0.977
Section 3		
Student Satisfaction and Self-Confidence in Learning	Two parts of the questionnaires were satisfaction (5 items) and self-confidence (8 items), 5-point Likert scales ranging from 1 (strongly disagree) to 5 (strongly agree), and satisfaction and self-confidence range 5–25 and 8–40, respectively. A higher score indicates that participants were highly satisfied /self-confident with simulation-based learning. Demonstrated reliability and validity [31].	0.883, 0.925

### Data analysis

Data were analyzed using SPSS version 27. Descriptive statistics were presented as mean and standard deviation (SD) for normally distributed variables. Linear correlation between two variables measured on the same interval or ratio scale was assessed using Pearson’s correlation coefficient (*r*). Multiple linear regression analysis was performed to control for other factors that may affect students’ satisfaction and self-confidence in antenatal simulation-based learning. Factors, including participant factors (cumulative grade point average (GPA), attitude toward SBE, professional identity, and perceived stress), facilitator factors (teaching competencies), and simulation design factors (attitude toward simulation design and perceived important design), were analyzed as independent variables in the regression analysis. Statistical significance was set at a level of 0.05. For qualitative analysis, content analysis was performed on verbatim transcriptions of the recorded interviews, which averaged 60 min each. After the interviews, field notes were recorded to document initial impressions. Data analysis was performed by three members of the research team (KK, SN, and AR) who had various perspectives on simulation-based learning. The researchers worked individually on the transcripts, which were read line-by-line and coded to identify key concepts. Smaller codes were grouped into larger categories, and these categories were grouped into major themes. A concurrent data collection and analysis strategy was used to explore new concepts in subsequent interviews in detail [32].

## Results

### Demographic and variable data in the quantitative results

**Fig. 1:**
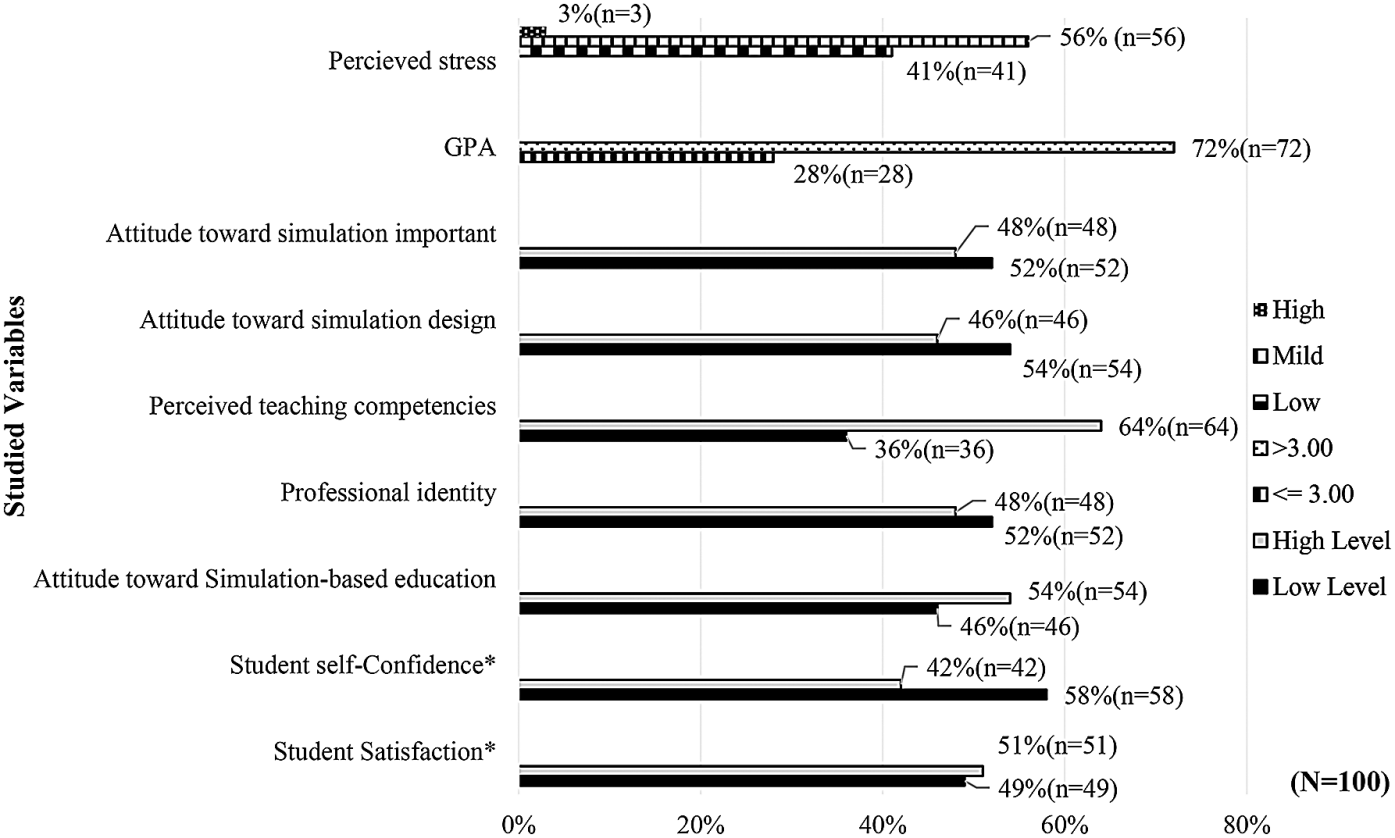
Number and percentage of participants categorized by personal, facilitator, simulation design factor, and studied outcomes

A total of 100 undergraduate nursing students were included in this study. The students’ age was between 20 and 26 years (mean = 21.43, SD = 0.82). More than 90% of the students were female. The independent and dependent variables were presented as percentages and separated into categories in Fig. 1. The cutoff values were determined as the mean of each variable in Table 2. Learner satisfaction and self-confidence were treated as dependent variables and demonstrated high levels with antenatal SBE. The mean satisfaction score was 20.55 (SD = 3.17, range 7–25), which showed that the participants were highly satisfied with SBL. Furthermore, participants were very self-confident in SBL, as indicated by the mean self-confidence score of 32.44 (SD = 4.76, range 16–40).

**Table 2:** Satisfaction and self-confidence in antenatal SBE according to personal, facilitator, and simulation design factors

Factors	Mean (SD)	Range	Satisfaction		Self-confidence	
			*r*	*p*	*r*	*p*
Personal factors						
GPA	3.22 (0.34)	2.41–3.90	−0.01	0.93	−0.03	0.73
Attitude toward SBE Low (< 76.07) High (≥ 76.07)	76.07 (9.10) 67.83 (5.82) 83.09 (4.09)	51.00–90.00 51.00–75.00 77.00–90.00	0.60	< 0.001*	0.53	< 0.001*
Professional identity Low (< 54.61) High (≥ 54.61)	54.61 (8.97) 47.44 (4.87) 62.37 (5.30)	34.00–75.00 34.00–54.00 55.00–75.00	0.24	0.01*	0.29	< 0.001*
Perceived stress Low stress (0–13) Mild stress (14–26) High stress (27–40)	14.94 (5.98) 9.29 (3.20) 18.34 (3.31) 28.67 (1.53)	0.00–30.00 0.00–13.00 14.00–26.00 27.00–30.00	−0.26	0.01*	−0.29	< 0.01*
Facilitator factors						
Perceived teaching competencies Low (< 24.63) High (≥ 24.63)	24.63 (3.05) 21.31 (2.76) 26.50 (0.69)	14.00–27.00 14.00–24.00 25.00–27.00	0.37	< 0.001*	0.47	< 0.01*
Simulation design factors						
Attitude toward ANC simulation design Low (< 78.40) High (≥ 78.40)	78.40 (11.16) 70.11 (7.51) 88.13 (5.32)	52.00–95.00 52.00–78.00 79.00–95.00	0.48	< 0.001*	0.52	< 0.001*
Attitude toward simulation importance Low (< 81.42) High (≥ 81.42)	81.42 (11.75) 71.71 (7.37) 91.94 (3.81)	51.00–95.00 51.00–81.00 83.00–95.00	0.29	0.00*	0.46	< 0.001*

*p value < 0.05; GPA, Grade Point Average

### Correlation for the satisfaction and self-confidence level in antenatal SBL according to personal, facilitator, and simulation design factors during the COVID-19 pandemic

Table 2 shows personal, facilitator, and simulation design factors related to satisfaction and self-confidence levels in antenatal simulation. A significant relationship was observed between personal, facilitator, and simulation design factors and students’ satisfaction. Attitude toward SBE (*r* = 0.601, *p* < 0.001), professional identity (*r* = 0.244, *p* = 0.01), perceived stress (*r* = − 0.255, *p* = 0.01), teachers’ competencies (*r* = 0.365, *p* < 0.001), attitude toward antenatal care (ANC) simulation design (*r* = 0.484, *p* < 0.001), and attitude toward simulation importance (*r* = 0.285, *p* = 0.00) were positively correlated with students’ satisfaction. Attitude toward SBE (*r* = 0.534, *p* < 0.001), professional identity (*r* = 0.292, *p* < 0.001), perceived stress (*r* = − 0.293, *p* < 0.01), teachers’ competencies (*r* = 0.468, *p* < 0.01), attitude toward simulation design (*r* = 0.519, *p* < 0.001), and attitude toward simulation importance (*r* = 0.457, *p* < 0.001) were significantly correlated with students’ self-confidence. However, no correlation was observed between cumulative GPA and students’ satisfaction and self-confidence in antenatal SBL.

### Predictive factors for students’ satisfaction with antenatal SBL during the COVID-19 pandemic

Table 3 shows personal, facilitator, and simulation design factors associated with students’ satisfaction with antenatal SBL during the COVID-19 pandemic. The regression analysis revealed that attitude toward SBE, a personal factor, was significantly associated with a high level of satisfaction with antenatal SBL (Beta = 0.473, *t* = 5.376, *p* < 0.001). Additionally, attitude toward ANC simulation design, a simulation design factor, was significantly associated with a high level of satisfaction with ANC simulation learning (Beta = 0.338, *t* = 2.611, *p* < 0.011). The multiple linear regression model accounted for 44.0% of the variance in satisfaction with ANC simulation learning (adjusted *R*^2^ = 39.0%).

**Table 3:** The personal, facilitator, and simulation design factors affecting students’ satisfaction with antenatal SBL

Factors	B	SE	Beta	t	p	95% CI
Personal factors						
GPA	−0.564	0.757	−0.062	−0.745	0.458	−2.069–0.940
Attitude toward SBE	0.165	0.031	0.473	5.376	< 0.001*	0.104–0.225
Perception of professional identity	0.010	0.031	0.028	0.314	0.754	−0.052–0.072
Perceived stress	−0.021	0.048	−0.040	−0.437	0.663	−0.117–0.075
Facilitator factors						
Perceived teaching competencies	0.025	0.110	0.024	0.227	0.821	−0.194–0.244
Simulation design factors						
Attitude toward ANC simulation design	0.096	0.037	0.338	2.611	0.011*	0.023–0.169
Attitude toward simulation importance	−0.032	0.030	−0.118	−1.052	0.295	−0.092–0.028

*R* = 0.66; *R*^2^ = 0.44; adjusted *R*^2^ = 0.39

B = beta coefficient; CI = confidence interval; SE = standard error; *t* = *t*-score of a regression model

*p value < 0.05

### The predictive factors of self-confidence in antenatal SBL during the COVID-19 pandemic

Table 4 shows personal, facilitator, and simulation design factors associated with students’ self-confidence in antenatal SBL during the COVID-19 pandemic. The regression analysis revealed that only attitude toward SBE was significantly associated with a high level of self-confidence in antenatal SBL (Beta = 0.331, *t* = 3.773, *p* < 0.001). The multiple linear regression model accounted for 45.0% of the variance in self-confidence in ANC simulation learning (adjusted *R*^2^ = 40.0%).

**Table 4:** The personal, facilitator, and simulation design factors affecting self-confidence in antenatal SBL

Factors	B	SE	Beta	t	p	95% CI
Personal factors						
GPA	−1.962	1.134	−0.144	−1.730	0.087	−4.213–0.290
Attitude toward SBE	0.173	0.046	0.331	3.773	< 0.001*	0.082–0.264
Perception of professional identity	0.061	0.047	0.116	1.316	0.191	−0.031–0.154
Perceived stress	−0.012	0.072	−0.014	−0.159	0.874	−0.155–0.132
Facilitator factors						
Perceived teaching competencies	0.201	0.165	0.129	1.215	0.228	−0.128–0.529
Simulation design factors						
Attitude toward ANC simulation design	0.068	0.055	0.159	1.233	0.221	−0.041–0.177
Attitude toward simulation importance	0.073	0.045	0.180	1.614	0.110	−0.017–0.163

*R* = 0.67; *R*^2^ = 0.45; adjusted *R*^2^ = 0.40

B = beta coefficient; CI = confidence interval; SE = standard error; *t* = *t*-score of a regression model

*p value < 0.05

### Qualitative results

A total of 20 third year nursing students participated in four focus group interviews. The participants’ age range was 20–24 years, and the average age was 21.55 years (SD = 0.99). The GPA ranged from 2.55 to 3.69 (average 3.20, SD = 0.32). Most participants were female (*n* = 18, 90%). Learner satisfaction scores ranged from 14 to 25 (average 20.75, SD = 4.20). Four major themes were generated, including positive attitude toward antenatal simulation, turning reassurance into confidence, I am really happy to learn, and being a good nurse motivates and stresses me.

### Theme 1: positive attitude toward antenatal simulation

Regarding antenatal nursing practices, the simulation-based design was implemented to enhance some of the essential competencies for nurses, such as perinatal history assessment and physical examination, advice for resolving common problems in each trimester of pregnancy, and abdominal examination in pregnancy. The students had a positive attitude toward prenatal simulation. They stated that antenatal simulation could increase their confidence in necessary skills, allow them to make mistakes and correct them, learn the correct practical techniques, prepare them for performing tasks in the ANC clinic, help practice complex cases, and gain patients’ trust. For example, a student disclosed her positive experiences after learning about perinatal history assessment with standardized patients. She learned the step by step process of assessment.

“I think simulation could help me learn step by step correctly after the teacher’s debrief. I think I learn a lot from all situations.” (a student in group 3).

Another student reported that the advantage of simulation is that it allows them to practice several times without putting patients at risk.

“The strengthening points of simulation from my viewpoint were that they allowed me to make something wrong and be able to fix it in the next round. In addition, it could reduce my nervousness in a real-life situation when I approach pregnant women.” (a student in group 2).

### Theme 2: turning reassurance into confidence

Many students stated that they received favorable feedback from instructors after simulation learning. Instructors used comments to improve the next set of scenarios. Students who participated in prenatal simulation labs might gain confidence in implementing these procedures in the ANC clinic. For example, a student stated that her antenatal competence improved after completing numerous simulations. She was able to learn and correct her practice and behaviors with the guidance and support of the teacher.

*““I believe I have continued to improve my antenatal care competencies. In simulation labs, I practice roughly 3–4 scenarios for which I know the correct advice patterns. When I go to the ANC clinic, I feel comfortable offering advice and performing abdominal examinations on pregnant women.” (a student in group 1)”*.

Another student stated that she was impressed by her instructor’s comments. Her instructor generously encouraged her without placing any undue pressure throughout the simulation practices. This instructional style may increase students’ confidence and help them perform better.

“When I used incorrect abdominal examination techniques and educated pregnant women in simulation, my instructor did not blame me. She gave me helpful counsel and discussions for finding a solution. It’s a pleasure to learn from her…” (a student in group 3).

### Theme 3: I am really happy to learn

Most students stated that they were happy to learn and practice through antenatal simulation. They reported four factors that influenced their positive emotions during the learning process, including teacher personalities (e.g., unpressured and kind), teaching techniques (e.g., positive reinforcement, positive feedback, and unlimited repetition), standard equipment (adequate), and learning environment (peer). For example, a student stated that she enjoyed learning with the ANC simulation because of the friendly instructor and cheerful teaching manner. She felt comfortable speaking with the teacher.

*““I feel this is the most happiness with learning in my nursing student life. I love her [teacher] teaching style. She was not a stressful person. Always, she provides vital points that hit the points of nursing care for pregnant women. I dare [I am confident] and am comfortable discussing with her.” (a student in group 4)”*.

“*The teachers’ praise is very important in influencing my study intention and increasing my daring [confidence] to practice*.” *(a student in group 2)*.

Another student described his favorite teaching method, which allows peers to give feedback after the simulations. He felt that he could learn from peer support as well.

“I favor my friends who are observers and then give feedback to me. Also, I can observe and give feedback to them as well. It meant that I could learn many cases; we could learn together and fix the weak points in the next cases.” (a student in group 2).

However, students stated that they perceived failure when they received negative feedback. For example, a student expressed her negative experiences and was unhappy to learn.

“If teachers blame us for doing things the wrong way or emphasize our faults rather than solving methods. It’s very bad and makes me very fail and don’t want to learn.” (a student in group 1).

### Theme 4: being a good nurse motivates and stresses me

Many students discussed the outcomes of ANC simulation learning, including gaining competencies to give pregnant women accurate, appropriate, and safe nursing care. Other students recognized that their personal and teachers’ expectations could put pressure on them and prevent them from implementing these skills in the real world because they were afraid of making a mistake. For example, a student expressed her expectation that ANC simulation learning allows nursing students to provide correct care, which makes pregnant women trust them.

“I would like to provide the correct care and be able to advise my patients. In addition, I need to receive patients’ trust. It’s quit [a lot of] pressure” (a student in group 4).

Moreover, a student reported that she would like to practice in an ANC simulation clinic several times because it would increase the quality of nursing care provided in the clinic.

“I hope when I have to practice in an ANC clinic, I will give them [pregnant women] the correct and safe care. I hope I will reduce my nervousness and have consciousness.” (a student in group 2).

## Discussion

During the COVID-19 pandemic, simulation learning was developed and implemented as a teaching modality. Nursing is an occupation that requires nursing students to build psychomotor, behavioral, and cognitive skills [33]. Simulation can be an effective learning experience that increases students’ knowledge and self-confidence and enables the development of clinical decision-making skills [17, 34]. However, satisfaction and self-confidence are the main learning outcomes obtained through simulation [35]. The study results showed that the personal and simulation design factors increased satisfaction and self-confidence in antenatal simulation learning among nursing students.

Attitude toward SBE was a strong significant predictor of students’ self-confidence in antenatal simulation learning. Moreover, the qualitative results showed how students developed their confidence in simulation learning based on teachers’ positive feedback, which allowed them to resolve and remediate skills. In this study, students expressed positive attitudes toward SBE, showing that simulation helped them prepare themselves for practice in the ANC clinic and practice complex cases that would benefit them in the future. However, previous studies have reported several negative opinions from students regarding SBL, including limited equipment/resources, inadequate realistic scenarios reflecting clinical settings, and inadequate space to practice simulation [36–38]. Furthermore, students have expressed negative emotions such as stress and anxiety [39]. Thus, it is important to have a positive and nurturing environment and appropriate teaching techniques to increase positive attitudes toward SBE, which strongly impact students’ confidence in learning.

In this study, attitude toward SBE and attitude toward simulation design were strong predictors of students’ satisfaction. Furthermore, many students expressed their satisfaction as a result of the teaching characteristics, simulation design, and positive learning process. Ross et al. [40] reported that undergraduate nursing students who completed SBL before their clinical practice were satisfied with this education. Additionally, nursing students benefited patients, they were accepted by clinical nurses, and their level of knowledge increased with this education. The simulation design and activities should be based on learners and their needs [41]. Bagnasco et al. [42] reported that satisfaction levels were related not only to available materials, instruments, and interactive simulations but also to the trainer’s expertise, approachability, and communication skills. A learning environment that promotes students’ satisfaction enhances motivation to study and increases the chance of meeting expected learning outcomes [43]. Therefore, the competence of trainers to meet learners’ needs and promote learner engagement should be considered when implementing SBL.

In this study, the qualitative analysis showed that some students accepted that they experienced stress during simulation learning. Experiencing high stress and anxiety levels during practice can decrease concentration in the simulation scenario [44]. Research studies have reported different causes of learners’ stress. The simulation may cause high stress and anxiety levels because of unfamiliar learning approaches [45]. Willhaus et al. [46] showed that nursing students could have negative experiences, such as stress and anxiety, which are often unexpected consequences of the simulation-based practice. The outcomes and expectations for each learning simulation should be clarified, and the level of difficulty should be appropriate for the students.

This study has a limitation. Since the data collection was based on self-administered online surveys where participants were asked about their past simulation learning experience, recall bias may exist. However, a strength of this study lies in its use of a mixed-methods research design, which may reduce self-reported bias [47] and strengthen the analysis, resulting in comprehensive research outcomes and a better understanding of the learners’ experiences. Furthermore, it serves as a reflection of the impact of instructional management under the constraints of the COVID-19 situation, particularly with limited practical training opportunities. This provides insights into potential paths for the future development of the antenatal SBL training practicum. In future research, it is advisable to design an experimental study that ensures effective outcomes, with a particular focus on assessing the potential impact of antenatal SBL on critical thinking skills. Furthermore, this study provides guidance for practical curriculum design in nursing education and practice, particularly regarding effective antenatal SBL. These implications encompass various aspects, including the preparation process aimed at fostering positive student attitudes toward SBE, enhancing teachers’ competencies in training, designing simulations to cover a range of skills and outcomes to boost student confidence, expanding to promote critical thinking skills, and addressing the psychological well-being of students during implementation.

## Conclusions

This study showed that GPA, attitude toward SBE, professional identity, perceived stress, teaching competencies, attitude toward simulation design, and attitude toward simulation importance influenced students’ satisfaction and self-confidence in antenatal SBL. Furthermore, attitude toward SBE and attitude toward simulation design were strong and significant predictors of student satisfaction, whereas attitude toward SBE was the only predictor of student self-confidence. Based on the qualitative analysis, four major themes were identified, including positive attitude toward antenatal simulation, turning reassurance into confidence, I am really happy to learn, and being a good nurse motivates and stresses me. The study results may contribute to the development of learning methods that enhance the effectiveness of antenatal SBL and ensure that nursing students achieve optimal benefits.

## Data Availability

The datasets used during the current study are available from the corresponding author on reasonable request.
